# Genome-Wide HMG Family Investigation and Its Role in Glycoalkaloid Accumulation in Wild Tuber-Bearing *Solanum commersonii*

**DOI:** 10.3390/life10040037

**Published:** 2020-04-10

**Authors:** Clizia Villano, Vincenzo D’Amelia, Salvatore Esposito, Maria Grazia Adelfi, Felice Contaldi, Rosalia Ferracane, Paola Vitaglione, Riccardo Aversano, Domenico Carputo

**Affiliations:** 1 Department of Agricultural Sciences, University of Naples Federico II, Via Università 133, 80055 Portici, NA, Italy; clizia.villano@unina.it (C.V.); adelfi.mariagrazia@gmail.com (M.G.A.); rosalia.ferracane@unina.it (R.F.); paola.vitaglione@unina.it (P.V.); carputo@unina.it (D.C.); 2 National Research Council of Italy, Institute of Biosciences and Bioresources (CNR-IBBR), Via Università 133, 80055 Portici, NA, Italy; Vincenzo.damelia@ibbr.cnr.it; 3 CREA Research Centre for Vegetable and Ornamental Crops, Via Cavalleggeri 25, 84098 Pontecagnano Faiano, SA, Italy; salvatore.esposito01@gmail.com (S.E.); felice.contaldi@gmail.com (F.C.)

**Keywords:** *HMG1*, wild potato, sterols, transgenic potato

## Abstract

Steroidal glycoalkaloids (SGAs) are a class of nitrogen-containing glycosides occurring in several plant families and biosynthesized through a specific pathway. HMG-CoA reductase is the first enzyme of this pathway, and its transcription can be regulated by biotic and abiotic stressors and even in a tissue-specific manner. This study aimed to characterize the *HMG* genes family in a tuber-bearing potato species, *Solanum commersonii*, using transcriptional and functional approaches. Our results provided evidence that four *ScHMGs* with different tissue-specificities represent the *HMG* gene family in *S. commersonii* and that they originated from *ScHMG1* through segmental duplications. Phylogenetic analysis suggests that *ScHMG1* is the direct ortholog of *AtHMG1*, which is associated with SGAs accumulation in plants. Its overexpression in *S. commersonii* revealed that this gene plays a key role in the accumulation of glycoalkaloids regulating the production of dehydrocommersonine.

## 1. Introduction

Steroidal glycoalkaloids (SGAs) are a class of nitrogen-containing glycosides occurring in several members of the plant family Solanaceae, such as tomato, eggplant and potato. They are toxic to humans and have putative roles in plant defense against environmental stressors [1]. SGAs can be produced in all parts of the plants, including the edible ones, such as eggplant and tomato fruits and potato tubers, as well as in leaves, flowers and roots. Nowadays, over 50 SGAs have been identified. Among them, trisaccharides α-solamargine and α-solasonine predominate in eggplants; tetrasaccharides α-tomatine and dehydrotomatine in tomatoes; and trisaccharides α-chaconine and α-solanine in potatoes [2]. In particular, α-chaconine and α-solanine comprise more than 90% of the total SGA content in the tuber [3]. It has been demonstrated that wild tuber-bearing potatoes can produce additional glycoalkaloids, and it has been postulated that, during domestication, Solanaceae selected only two predominant SGAs, in order to have a lower number of SGAs, while maintaining resistance against phytopathogens [2]. Indeed, depending on the amount and type, SGAs can trigger various effects on plant physiology, and their role can be critical for plant resistance to biotic stressors, including fungi, bacteria, viruses and insects [2]. For example, SGAs in potatoes cause toxicity to the Guatemalan potato moth and the Colorado potato beetle, some of the worst potato pests [4,5]. Plant SGAs are biosynthesized by a specific pathway, where the major player is the 3-hydroxy-3-methylglutaryl coenzyme A reductase (HMG-CoA), which catalyzes the first committed step of sterol precursors biosynthesis. Once formed, the precursors undergo several biochemical processes, such as hydroxylation, oxidation, transamination and glycosylation, before they produce SGAs [6]. The HMG-coding genes are highly regulated by both abiotic and biotic stressors, like light [7], hormones [8], wounding [9] and pathogens [10]. At the genome level, *HMG* is encoded by small gene families, with two genes characterized in *Arabidopsis thaliana* [11] and radish [12], three in *Hevea* [13] and four in tomato [14]. In the cultivated potato *Solanum tuberosum*, three *HMGs* genes have been characterized [15]. It has been demonstrated that they are differentially expressed in response to wounding (*StHMG1*) or pathogens (*StHMG2* and *StHMG3*) [7]. Krits et al. [16] showed that there is a correlation between transcript levels of *StHMG1* and total SGAs. However, to the authors’ best knowledge, no studies have examined HMG gene family members in wild potato relatives, which can biosynthesize a greater amount of glycoalkaloids when compared to the cultivated form [17,18]. Such a lack of studies contrasts with the interest in efficiently using wild potatoes in breeding programs to obtain varieties with improved traits. Among wild tuber-bearing species, *Solanum commersonii* has become a model for molecular biology, genetics and breeding, garnering significant research interest. Indeed, this species possesses several desired resistances (to nematodes, bacteria and viruses) lacking in the cultivated potato [19,20,21,22]. It is particularly attractive for its freezing tolerance and capacity to cold acclimate [23]. Furthermore, it is the first wild potato whose genome sequence has been released [23], paving the way to different studies aimed to gain insights into genomic evolution at the sequence level from the wild to the cultivated form [24,25]. *S. commersonii* possesses a diverse mixture of glycoalkaloids, such as dehydrotomatine, dehydrodemissine and dehydrocommersonine, which are among the most poisonous compounds against different potato pests [26]. In this species, Vazquez et al. [27] also reported the presence of demissine, commersonine and a-tomatine. All of these characteristics make *S. commersonii* a suitable material for the elucidation of the role of HMGs in glycoalkaloid synthesis in potatoes and for studying the evolution of the HMG gene family from wild to cultivated species. Here, we report for the first time, in a wild potato, a study on the *HMG* gene family and the functional analysis of the gene *ScHMG1* encoding HMG-CoA reductase.

## 2. Materials and Methods 

### 2.1. Identification of Candidate HMG Genes and Phylogenetic Analysis

We used as queries the known HMG protein sequences of *A. thaliana* (downloaded from phytozome.jgi.doe.gov/pz/portal.html [11]) to search the orthologs in *S. commersonii* clone cmm1T of PI243503 (hereafter called cmm1T), *S. tuberosum* Group Phureja (DM1-3 516 R44) and *S. lycopersicum*, using Blastp with a threshold for significance of at least 10^3^, in order to consider a match. Protein sequences of putative HMG in tomatoes and potatoes were confirmed through domain analysis by using the online software NCBI Search Domain [28]. All confirmed candidates were then aligned by using MUSCLE implemented in MEGAX [29], and the resulting matrix was used as input to identify the best fit for the substitution model for phylogeny (option find best DNA/Protein model). The phylogenetic analysis was finally performed, using the maximum likelihood method, with the best-predicted option JTT+G and 1000 bootstrap replicates. Our candidates were named based on the nomenclature of *A. thaliana* genes and the phylogenetic analysis. To further investigate the evolutionary history of our candidate proteins, the exon–intron organization was also studied by the online Spidey program [30]. The expression profiles of the *HMG* genes across four different tissues of *S. commersonii* and *S. tuberosum* (flower, leaf, root and stolon) were also studied. In particular, raw data for *S. commersonii* (deposited under the study SRP050412) and *S. tuberosum* (ERR029909, ERR029910, ERR029914 and ERR029917) were downloaded and analyzed, using Cufflinks–Cuffquant software (version 2.2.1); expression values for each gene were expressed as RPKM (Reads Per Kilobase of transcript per Million mapped reads), as described by Esposito et al. [24,31,32].

### 2.2. Determination of Gene Duplication Patterns

The patterns of HMG gene duplication were determined as segmental or tandem based on the position of paralogues on chromosomes, as reported by Zhao et al. [33] and Xia et al. [34]. The PGDD [35] was used to determine the genomic duplication blocks. The Ks values of paralogues in segmental duplication blocks were retrieved from the PGDD database computed by the KaKs calculator [36]. The timing of duplication events can be estimated by using the Ks value and a given clock-like rate, λ, through the formula T = Ks/2λ where, for tomato, λ = 1.5 × 10^−8^ substitutions per synonymous site per year [37]. 

### 2.3. Plant Material

Plantlets of *S. commersonii* were propagated in vitro on Murashige and Skoog (MS) medium (Sigma-Aldrich, St. Louis, MO, USA) with 1% (w/v) sucrose and 0.8% (w/v) agar. They were incubated at 24 °C, exposed to an irradiance of 200 mol m^2^ sec^1^ and under a 16 h/8 h (light/dark) photoperiod, as described by D’Amelia et al. [38].

### 2.4. Overexpression of ScHMG1 in S. commersonii

The *ScHMG1* coding sequences were cloned in the 35SCaMV expression cassette of pGWB411 [39], using Gateway recombination technology (Life Technologies, Carlsbad, CA, USA). *Agrobacterium tumefaciens* cells ELECTRO MAX LBA4404 (Life Technologies, Carlsbad, CA, USA) transformed with each expression vector were used for co-cultivation of cmm1T leaf explants, according to the protocol of Cardi et al. [40]. A single plant was taken from a single callus. Transgenic plantlets were verified by genomic PCR on kanamycin gene-specific primers (Table S1) as reported by Brulè et al. [41].

### 2.5. RNA Extraction and Gene Expression Analyses

Total RNA was extracted from transformed plantlets. RNA extraction was performed by using the Spectrum Plant Total RNA Kit (Sigma-Aldrich, St. Louis, MO, USA) and the On-Column DNase I Digestion Set (Sigma-Aldrich, St. Louis, MO, USA). Then, 1 μg of RNA was reverse transcribed, using Oligo-Dt (20) and the SuperScript III reverse transcriptase (Invitrogen, www.invitrogen.com) in 20 μL final reaction volume, according to the manufacturer’s instructions. The obtained cDNA was used to monitor the expression change of genes through RT-qPCR. The experiment was carried out by using the 2x QuantiFast SYBR Green PCR Master Mix (Qiagen, Valencia, CA, USA) with ABI PRISM 7900HT (Applied Biosystems, Foster City, CA, USA). Each 15 μL reaction contained 300 nM of each primer and 1:5 diluted cDNA. Cycle conditions indicated by QuantiFast SYBR Green PCR Kit handbook (Qiagen, Valencia, CA, USA) were used. The housekeeping gene used for normalization of expression data was adenine phosphoribosyltransferase (APRT) in transgenic plantlets expression analysis. Results were analyzed by using the ABI PRISM 7900HT Sequence Detection System Version 2.1 (Applied Biosystems, Foster City, CA, USA). The relative expression was estimated according to the ∆∆Ct method [42], as described by Villano et al. [43] and Di Meo et al. [44]. Three biological and three technical replicates were prepared for the analysis.

### 2.6. Copy Number Determination 

Quantitative real-time PCR (qPCR) was used to estimate transgene copy numbers in transgenic plants [45]. Genomic DNA was extracted from transformed plantlets, using the DNeasy Plant Mini Kit (Qiagen, Valencia, CA, USA), following the manufacturer’s guidelines. Then 10 ng of genomic DNA was used in a 12.5 μL final reaction volume, according to the manufacturer’s instructions. The experiment was carried out in triplicates, using the 2x QuantiFast SYBR Green PCR Master Mix (Qiagen, Valencia, CA, USA) with ABI PRISM 7900HT (Applied Biosystems, Foster City, CA, USA). Relative gene copy numbers were obtained by using the ΔCt method of relative quantification, since the genes were amplified with similar efficiency in each sample. The relative gene copy number was calculated as 2^−ΔCt^, where ΔCt = Ct_target_ − Ct_reference_. The single-copy gene GBSS1 (granule-bound starch synthase 1) was used as a reference gene.

### 2.7. SGA Determination 

SGA determination was performed by Liquid Chromatography–High Resolution Mass Spectrometry (LC–HRMS) analysis. Freeze-dried leaves (250 mg) were extracted with 20 mL of 2% acetic acid solution, using an Ultra Ika T18 basic Ultraturrax (Staufen, Germany) for 1 min. The extract was centrifuged at 14,800 rpm for 10 min and directly used for HMRS analysis. LC–HRMS data were acquired on an Accela U-HPLC system coupled to an Exactive Orbitrap mass spectrometer equipped with a heated electrospray interface (HESI) (Thermo Fisher Scientific, San Jose, CA). Chromatographic separation was performed, using a Gemini C18-110Å 5 μm column (150 mm × 2.0 mm) (Phenomenex, Torrance, CA) at 30 °C. The mobile phase consisted of 0.1% formic acid water (A) and 0.1% formic acid acetonitrile (B). Gradient elution was linearly programmed as follows: 10% B (1 min), 10%–90% B (7 min), constant to 90% B (2 min), 90%–10% B (2 min). The solvent flow rate was 200 μL/min, and the injection volume was 10 μL. The acquisition was performed in positive ionization mode in the mass range of *m/z* 100–1200. The resolving power was set to 50,000 full width at half-maximum (FWHM, m/z 200), resulting in a scan time of 1 s. The automatic gain control was used in balanced mode (1 × 10^6^ ions); the maximum injection time was 100 ms. The spray voltage was at 3 kV, the capillary voltage was 30 V, the capillary temperature was at 275 °C and a sheath and auxiliary gas flow of 30 and 15 arbitrary units were used. The instrument was externally calibrated in the positive ion mode, following the Thermo Exactive calibration procedure. Chromatographic data acquisition and peak integration were performed by using Xcalibur software (Thermo Fisher Scientific, San Jose, USA). Compounds’ identification was performed by using exact mass values up to the fifth decimal digit, with mass tolerance ± 5 ppm. All analyzed compounds were expressed as equivalents of α-solanine. Molecular formula, theoretical mass, experimental mass and mass accuracy are reported in Table S2.

## 3. Results and Discussion

### 3.1. Comparative and Phylogenetic Analysis of the HMG Gene Families in Solanaceae 

Several lines of evidence support that HMG-CoA reductase (HMG) has a key role in the regulation of the metabolic flux through the plant sterol biosynthetic pathway. Here, a genome-wide study of the HMG family has been carried out in three Solanaceae species, and one of these genes, *ScHMG1,* has been functionally characterized in wild tuber-bearing *S. commersonii.* Our study began with the identification of the *HMG* gene family members in cultivated tomato *S. lycopersicum,* cultivated potato *S. tuberosum* and *S. commersonii*, starting from the genome of the model plant *A. thaliana.* To date, two differentially expressed *HMG* genes, *AtHMG1* and *AtHMG2,* have been characterized in *A. thaliana* [11,46]. Their contrasting role in SGA biosynthesis and plant development has been largely demonstrated. For example, the analysis of a null *AtHMG1* evidenced the essential role of this gene in controlling several plant developmental traits, such as dwarfism, senescence and sterility. By contrast, the silencing of *AtHMG2* did not affect either the phenotype or the fertility of the plant under normal growth conditions [47]. 

**Figure 1:**
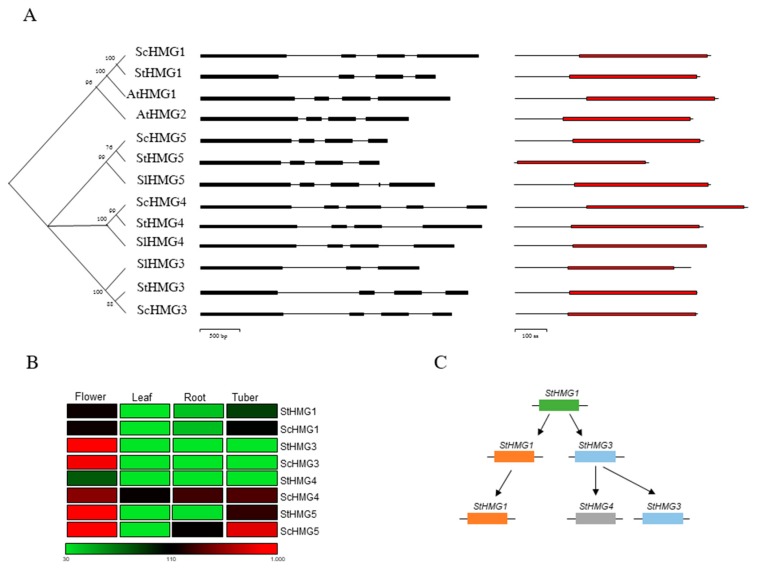
(**A**) Phylogenetic analysis of the *HMG* gene family in *Arabidopsis, Solanum lycopersicum, S. tuberosum* and *S. commersonii*. Domain distribution (red boxes represent the HMG-CoA reductase class I domain) with NCBI search domain and exon–intron structure (black boxes represent the exons) of HMGs from *Arabidopsis*, *S. lycopersicum*, *S. tuberosum* and *S. commersonii*. (**B**) Expression patterns of *ScHMGs* and *StHMGs* in flowers, leaves, roots and tubers. (**C**) Duplication events occurring in *HMG* genes in *S. tuberosum*.

Within *Solanaceae*, recently, Zhang et al. [7] reported that the *S. tuberosum* genome holds two *HMG* genes (*StHMG1* and *StHMG2*). However, in our study, we were able to identify four *StHMGs*, namely *StHMG1, StHMG3, StHMG4* and *StHMG5* (Figure 1A), and here we propose a new potato *HMG* gene family classification. Direct orthologs of *AtHMG1* were found in *S. tuberosum* (PGSC0003DMP400017516) and *S. commersonii* (maker_scaffold1783_augustus_gene_0_28), but not in tomato. By contrast, no robust orthology relationship was found for *AtHMG2,* which did not cluster with any *Solanum HMGs* (Figure 1A). The remaining sequences were named *HMG3*, *HMG4* and *HMG5*, using progressive numbering. It is noteworthy that *StHMG5* has been previously annotated as *StHMG2* [26,48]. However, our data rule out any orthologous relationship between *StHMG5* and *AtHMG2*. Regarding *StHMG4*, we found two different isoforms in *S. tuberosum* annotated as PGSC0003DMP400024173 (which has a strong similarity with the N-terminal portion of *SlHMG4* and *ScHMG4*) and PGSC0003DMP400024174 (strongly similar to the C-terminal portion of the same proteins). Therefore, we considered them to be a single protein. *HMG3*, *HMG4* and *HMG5* had one-to-one direct ortholog in *S. commersonii*, *S. tuberosum* and *S. lycopersicum,* suggesting a gene expansion occurred in *Solanaceae* since it last shared a common ancestor with the model species *A. thaliana.*

We identified four *HMG* genes also in *S. commersonii*, with one-to-one orthologs to the *StHMGs*. They were therefore named *ScHMG1, ScHMG3, ScHMG4* and *ScHMG5*. No direct orthologs to *AtHMG2* were identified. To gain insights into the evolution of *HMG* genes between *Arabidopsis*, *S. lycopersicum, S. tuberosum* and *S. commersonii*, we examined their exon–intron structure (Figure 1A). Most of the genes showed four exons, with the only exception being *SlHMG3* (three exons), *SlHMG4* and *ScHMG4* (five exons) (Figure 1A, Table S3). At the protein level, a single common HMG-CoA reductase class I domain was found in all orthologous sequences; this domain consists of five features: catalytic residue, NADP(H) binding site, substrate-binding pocket, inhibitor binding site and tetramerization interface. Both *Arabidopsis* and the two potato species’ HMGs showed all these features, whereas SlHMG3 contained only the NADP(H) binding site and tetramerization interface (Table S3). Recently, Zhang et al. [7] reported that the potato *HMG* genes (*StHMG1* and *StHMG2*) could be differently regulated in potato tubers subjected to light exposure, demonstrating a different specialization of these genes in response to abiotic stressors. Even in *Arabidopsis*, a different specialization of HMGs in the induction of SGAs has been evidenced [49]. Hence, we explored the expression of the different HMG genes, using public RNA-seq data and also focused on the divergence between *S. commersonii* and *S. tuberosum*. First, the gene structure previously described was confirmed for all *HMG* genes, excluding the possibility of any pseudogene in the dataset. Then, *HMGs* of both species were expressed in flower, leaf, root and tuber tissues (Figure 1B). To some extent, a match of the *HMG* expression patterns between *S. commersonii* and *S. tuberosum* and across all the analyzed tissues was found. In particular, most of the *HMGs* were highly expressed in flower, while a higher specificity of *HMG5* to tuber tissue was found in both species. *HMG1* and *HMG3* paralogs in *S. commersonii* and *S. tuberosum* showed a similar expression pattern. We observed a strong expression of *ScHMG4* throughout all tissues, including in the leaf, where no other *HMGs* were particularly expressed. Overall, these results suggest a different plant-tissue-specificity and regulation of *HMGs*. *HMG-CoA reductase 1* is regulated at the transcriptional, posttranscriptional and posttranslational levels, and reduced activity is associated with reduced mRNA levels [50,51]. A high abundance of mRNA transcripts was observed in flowers, where different terpenoids usually accumulate [52]. *HMGs* expressed in the tubers are particularly interesting, since they may drive the production of glycoalkaloids in the edible part of the potato. In terms of expression profile, *S. commersonii* has two tuber-specific HMGs (*ScHMG4* and *ScHMG5*) compared to the cultivated potato, in which only *StHMG5* is highly expressed in tubers. This outcome correlates with the higher level of terpenoids and general glycoalkaloids of wild potato tubers compared to the cultivated potatoes. 

In this study, the relative contributions of tandem and segmental duplications of ScHMGs were investigated. For the former, we based the analysis on the chromosomal localization of HMG orthologs in *S. commersonii*, while for the latter, the Plant Genome Duplication Database (PGDD) was interrogated. However, since a specific repository for *S. commersonii* at the PGDD is not yet available, we retrieved the gene HMG IDs of *S. tuberosum*, along with their ks values. We hypothesized that the results harvested in potato reflect what occurs in *S. commersonii*. Even though tandem duplications were not found, two segmental duplications were identified. In particular, *StHMG3* was present both on segmental block_35 (ks 0,72; ks/ka 0,07) and on block_88 (ks 1; ks/ka 0,21)*,* suggesting that it amplified twice in potato. Notably, we found that *StHMG3* localized, along with *StHMG4* on block_35 and with *StHMG1* on block_88. Using ks values retrieved from the database and the molecular clock for potato, we showed that *StHMG3* originated from *StHMG1* by a segmental duplication event that occurred around 33 million years ago (MYA), whereas *StHMG4* originated later, from *ScHMG3* (~25 MYA) (Figure 1C). Our findings underly the idea that the number of *HMG* copies expanded through segmental duplication rather than other mechanisms, such as tandem duplication or retrotransposition.

### 3.2. ScHMG1 Overexpressing Plants and Their Expression Pattern 

**Figure 2:**
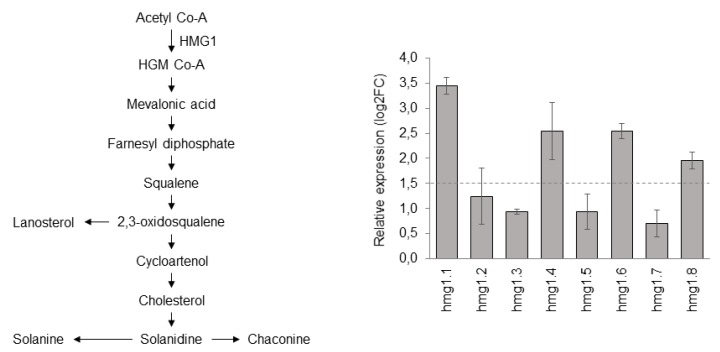
Schematic representation of the steroidal glycoalkaloids pathway and expression of *ScHMG1* in positive transgenic lines.

Based on our findings, among the four candidates identified in this study, we decided to use *ScHMG1* to test its role in the biosynthesis of SGAs in *S. commersonii*. Previous papers on tobacco demonstrated that the constitutive expression of HMG resulted in phytosterol overproduction, indicating a major role of HMG in the upregulation of sterol abundance [53,54]. Carpintero et al. [26] and Ginzberg et al. [51] provided evidence that *StHMG1* and *StHMG2* were the primary metabolism genes directly involved in SGA biosynthesis, and that overexpression of *StHMG1* in potato plants increased SGA content in the leaves. No papers have been published on the role of their orthologs in wild potato species, a strategic source of stress resistances and tuber quality traits. In the present study, we set up a constitutive overexpression of *ScHMG1* in *S. commersonii* and investigated both its expression and the abundance of SGAs in positive clones. Among all the HMGs found in *S. commersonii*, *ScHMG1* is the direct ortholog of the *Arabidopsis AtHMG1,* which has been found to play important physiological roles for plant growth and development [47,49,55]. After transformation, eight explants of *ScHMG1* were positive to genomic PCR, with a transformation efficiency of 8.9% (not shown). The transgene copy number was efficiently identified through qPCR. It revealed that four *ScHMG1* transgenic plants (hmg1.3, hmg1.4, hmg1.6 and hmg1.7) contained a relative gene copy number higher than 1 (data not shown). *ScHMG1* overexpression in leaves of transgenic plants, compared to the control plants, varied from 0.7 in hmg1.7 to 3.4 in hmg1.1 (Figure 2). Based on a threshold of 1.5 log2FC, we selected hmg1.1, hmg1.4, hmg1.6 and hmg1.8 transgenic lines for further analysis. 

**Figure 3:**
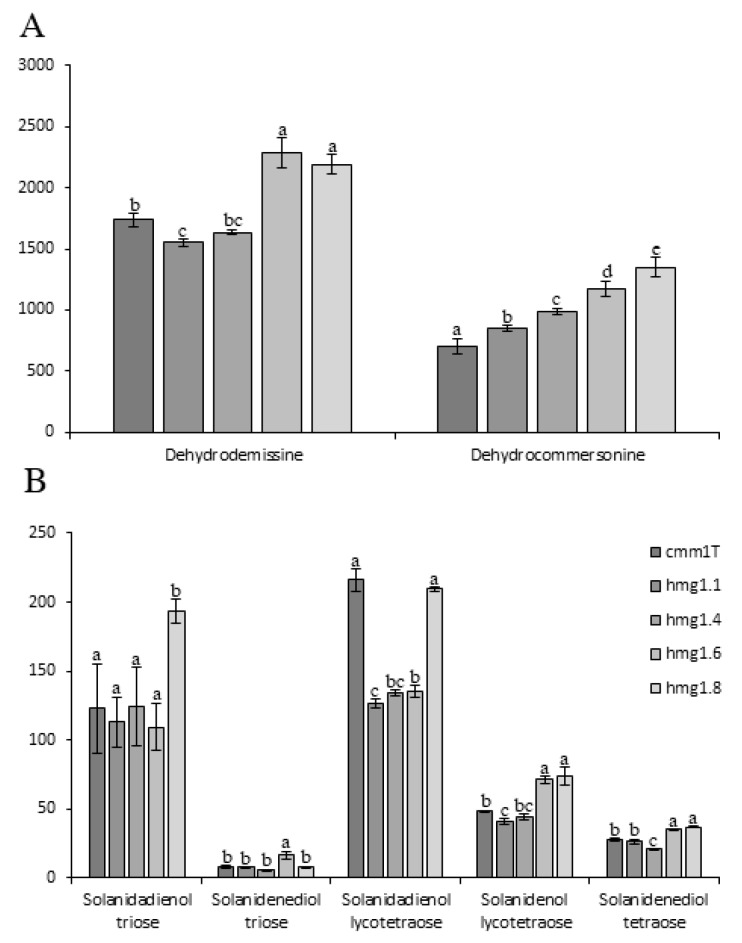
Major (**A**) and minor (**B**) glycoalkaloids content detected in leaves of wild type (cmm1T) and selected transgenic plants, hmg1.1, hmg1.4, hmg1.6 and hmg1.8. Each value represents the mean of three replicates (±SD). Means denoted by the same letter did not differ significantly at *p* ≤ 0.05, according to Duncan’s multiple range test.

The levels of major (dehydrodemissine and dehydrocommersonine) and minor (solanidadienol triose, solanidenediol triose, solanidadienol lycotetraose, solanidenol lycotetraose and solanidenediol tetraose) glycoalkaloids were determined in transgenic clones and in the untransformed control (cmm1T) (Figure 3A,B, respectively). 

Solanidenediol triose was the less abundant SGA in all samples, with an average of 9.2 (±3.7) mg/100 gr DM. Concerning major SGAs, dehydrocommersonine varied from 706.2 (cmm1T) to 1351.3 (hmg1.8) mg/100 gr DM, while dehydrodemissine ranged from 1551.9 (hmg1.1) to 2280.4 (hmg1.6) mg/100 gr DM. All transgenic lines had a dehydrocommersonine content significantly higher than that of the untransformed cmm1T. A different behavior was observed for minor SGAs, which did not exhibit a clear accumulation trend. Solanidadienol and solanidenediol triose content were significantly different than the control only in hmg1.8 and hmg1.6, respectively. Conversely, solanidadienol lycotetraose, solanidenol lycotetraose and solanidenediol tetraose contents were significantly different than the control in all transgenic lines, except for hmg1.8, hmg1.4 and hmg1.1, respectively. Our results are similar to those of Ginzberg et al. [51], who overexpressed HMG1 from *S. chacoense* in cultivated potatoes and did not find a consistent increase of SGA in transgenic lines. Furthermore, the expression levels of the ectopic *HMG1* reported by Ginzberg et al. [51] in potato were much higher than those of our study (about 20 times to 700 times higher). It can confer the additional hypothesis that the effect can be masked by the possibility of coordinated regulation of the downstream pathway. Krits et al. [16] and Ginzberg et al. [51] showed correlations between transcript levels of *StHMG1* and SGA production in *S. tuberosum* genotypes. In our work, we did not detect a consistent correlation between *ScHMG1* and total SGAs in leaf, except for dehydrocommersonine, which significantly increased in all our transgenic lines compared to cmm1T. Our results suggest two hypotheses: (1) In *S. commersonii* leaves, there is a more complex regulation which blocks the contribution of terpenoid precursors (produced by the activity of HMG-CoA) in SGA accumulation; (2) the *HMG1*-coded enzyme is weakly active, and consequently other HMG genes here identified can be stronger activators. This latter hypothesis is an important issue for future research aimed to investigate the contribution of the different *HMG* genes in SGA production.

## 4. Conclusions

SGAs are secondary metabolites that are associated with resistance to various environmental stresses in Solanaceae. Among the key genes of the SGA pathway, HMG plays a critical role and has been investigated in several cultivated plants. By contrast, no information is available on the behavior of HMG genes in wild species. In the present study, we proposed a new potato HMG gene family classification and showed that both cultivated *S. tuberosum* and its wild relative *S. commersonii* harbor four *HMG* genes. Our research suggests that HMG homologs arose from HMG1 through segmental duplication events rather than other mechanisms of gene duplication. *ScHMG1* is also the direct ortholog of *AtHMG1*, known to be a key gene in SGA regulation in *Arabidopsis*. *ScHMG1* was successfully overexpressed in *S. commersonii,* allowing the identification of a correlation between this gene and dehydrocommersonine accumulation.

## Supplementary Materials

The following are available online at https://www.mdpi.com/2075-1729/10/4/37/s1, Table S1. Name and sequence of primers used in the present study. Table S2: Molecular formula, theoretical mass, experimental mass and mass accuracy used in the HRMS analysis. Table S3. Characteristics of the *HMG* genes identified in the present study. For each gene, Gene and Sequence ID, protein sequence and length, gene structure (exon number and length and intron length) and the features of HMG-CoA reductase class I domain are reported.
